# Stressful life events during the perimenopause: longitudinal observations from the seattle midlife women’s health study

**DOI:** 10.1186/s40695-023-00089-y

**Published:** 2023-09-05

**Authors:** Annette Joan Thomas, Ellen Sullivan Mitchell, Kenneth C. Pike, Nancy Fugate Woods

**Affiliations:** 1 College of Nursing, Seattle University, Seattle, WA USA; 2 Office of Nursing Research, University of Washington, Seattle, WA USA; 3 Child, Family, and Population Health Nursing, University of Washington, Seattle, WA USA; 4 Biobehavioral Nursing and Health Informatics, University of Washington, Seattle, WA USA

**Keywords:** Midlife women, Stressful life events, Group-based trajectory models, Demographic characteristics, Menopausal transition, Socioeconomic factors, Seattle midlife women’s Health Study

## Abstract

**Background:**

Midlife is a time of increased responsibilities for women who have multiple roles including taking care of children, caring for elderly parents, managing households, and working outside the home. With little time for themselves, women additionally experience stressful life events (SLEs). The purpose of this study was to describe the longitudinal patterns of SLEs of women during midlife and to identify predictors of the SLE longitudinal patterns using baseline data of socio-economic factors and demographic characteristics.

**Methods:**

Women who were part of the Seattle Midlife Women’s Health Study (SMWHS), a longitudinal study spanning more than 23 years, who had SLEs measured at baseline and at years 2, 7, and 10 were included in these analyses (N = 380 women at baseline). The Life Event Scale (LES), a 70-item scale based on a yes/no response and a Likert-based scoring system with 0 (no effect) to 4 (large effect), was used to determine the total and impact scores of midlife women. The LES was adapted to midlife women from the Norbeck Scale for younger, pregnant women. Analytic strategies consisted of a group-based trajectory model (GBTM) to examine subgroups of women with similar exposure to SLEs using socio-economic factors (gross family income, education, race/ethnicity, employment), demographic variables (age, marital status, being a parent), and menopausal transition stage to differentiate trajectories over time.

**Results:**

Approximately 86% of women had medium high exposure to undesirable SLEs with a slight decrease (65.5%), or a sharp decrease (20.1%), over 10 years. The majority (approximately 64%) had moderate, sustained impact ratings, while approximately 35% had impact ratings that decreased over time. Most women (approximately 88%) reported desirable life events, which were sustained over the ten years, and which may help to balance or offset the high ratings of undesirable stressful life events. The rated impact of these desirable events decreased slightly over time for 65% of the sample. Socio-economic factors, demographic variables, and menopausal transition stages were not significant predictors of any of the four GBTMs.

**Conclusion:**

Midlife women experience SLEs throughout the menopausal transition. Most of these midlife women had had a large amount of sustained stress over 10 years although all trajectories decreased to some extent over time. Since the menopausal transition stages were not significant predictors of the ratings of SLEs, a more complex set of factors, including social as well as biological, may explain the ratings of the women over the course of this ten-year observational study.

**Supplementary Information:**

The online version contains supplementary material available at 10.1186/s40695-023-00089-y.

## Introduction

Studies of women’s experiences of stress during midlife have revealed the complexity of their lives as influential. Women’s responsibilities, such as caring for their children, working to generate income, caring for their older parents, being in a partnered relationship, dealing with healthcare issues of their own as they age, along with experiencing the menopausal transition contribute to stress perceptions.

Women’s descriptions of challenges they experience indicate that midlife is a stressful time, yet most studies have focused on daily stressors women encounter, assessing perceived stress with little emphasis on their experiences of major stressful life events. Perceived stress refers to the thoughts and feelings about one’s own daily experiences [1, 2] in contrast to stressful life events. Stressful life events (SLEs) are major events that expose people to traumatic or out-of-the-ordinary occurrences in a person’s life, such as death of a spouse or parent, divorce, imprisonment, dismissal from work, marriage, and/or retirement.

Nonetheless, studies of perceived stress ratings indicated that they decreased over time for most midlife women [3, 4] and progression through the menopausal transition stages had little effect on perceived stress. Instead, the context of midlife women’s lives, which includes juggling multiple roles and progressing through role transitions were most challenging. For example, women were coping with losses due to deaths, managing health challenges of their own, for some in the context of limited resources, e.g., financial hardship and having completed less education [5].

Recent investigations examining the role of structural racism in the United States have prompted examination of racial/ethnic associations with perceived stress. An earlier longitudinal analysis of perceived stress scores in the Study of Women’s Health Across the Nation (SWAN) population [3] indicated that Hispanic women from the New Jersey site reported higher perceived stress than any of the other racial/ethnic groups, including Black, White, Chinese, and Japanese women. In addition, those with financial hardship and lower levels of education rated their stress as greater [3]. The Seattle Midlife Women’s Health Study (SMWHS) participants who rated their stress levels higher were those who reported physical aging changes and poorer perceived health, inadequate income, lower social support, greater role burden, history of sexual abuse, depressed mood, and being employed. In a final multivariate model, employment, depressed mood, and perceived health were most influential. Owing to low levels of representation of African American and Asian American participants, the effect of race was not estimated in these analyses [4]. Changes in perceived stress have been associated with aging, yet midlife women experience changing life contexts related to their life roles, changing bodies, including changes related to the menopausal transition and general health, as well as the changing material conditions of their lives. Whether the relationship of these same factors to major life event stress is similar to those results reported above remains to be determined.

SLEs can be judged as undesirable or desirable and may have differing levels of impact. For example, the illness of a parent is an undesirable stressful life event while marriage is usually a desirable one. Both events may have varying levels of impact, ranging from no effect to a great effect depending on those who experience them.

Studying stressful life events women experience has been challenging owing to initial research on the topic focusing predominantly on populations of men or younger women. In the 1980s amid growing interest in studies of women, Norbeck developed the Life Event Questionnaire (LEQ) for the purpose of studying young adult women during the perinatal period [6]. Because the LEQ was tailored for young women, there were several major life events commonly reported by midlife women that were not included in the scale. Investigators for the SMWHS adapted the scale with Norbeck’s permission, referred to here as the Life Events Scale (LES), to include stressful life events commonly experienced by midlife women such as the death of a parent and the birth of a grandchild [7, 8].

Earlier analyses of data from the SMWHS examined undesirable life events among midlife women, revealing that Financial, Love and Marriage, and Family/Close Friend life event categories were reported most frequently [8], consistent with qualitative reports by women at the completion of the study [5]. Categories with the highest frequency changed by each time point or occasion of data collection during 10 years of follow-up: Financial events remained among the most frequent over all four time points, with Family/Close Friends and Crime/Legal matters rated among the highest for three time points. For the last two time points of data collection, Health and Personal/Social events (e.g., change in family gatherings, developed a new friendship) were among the highest frequency events. In contrast, events women rated as most undesirable and having the greatest impact over all timepoints of data collection included those in the Family/Close Friends category, and to a lesser degree Personal/Social, Health, and Work categories. Divorce, death of family members, being the victim of a violent crime, health problems, work problems, and relocating residences were among those individual events ranked with the highest impact (4 = great effect). As might be anticipated, age, income, marital status, being a parent, and reproductive aging stages were differentially associated with the categories of undesirable life events [8].

Although there have been earlier longitudinal reports of perceived stress during the menopausal transition and early post-menopause [3, 4] revealing that progressing through the menopausal transition stages had little effect on perceived stress, to date investigators have devoted little attention to the longitudinal patterns of stressful life events with the exception of results reported from the Study of Women and Health Across the Nation (SWAN) and the SMWHS. SWAN participants (N = 3044) completed the Psychiatric Epidemiology Research Interview Life Events Scale (PERI) annually over a 15-year period. The PERI categories, which included work/economic (including partner unemployment), relationship, family, legal/police problems, caregiving, bereavement, violent events to self/family, and illness/accident to loved ones, were examined in relationship to race, age, and education. The number of life events declined slightly in the SWAN population with age, reaching a plateau at older ages (at approximately 55–60 years). Racial differences were notable with Black women experiencing more life events than White women; and, Chinese, Hispanic and Japanese women experiencing fewer events. Although the patterns remained similar as women aged, Hispanic women reported fewer events as they aged until their early 50s when the number increased. There was an overall pattern of declining number of events as women aged for work problems, economic problems, and partner unemployment. When race was considered, Black women reported greater work- and finance-related events and smaller declines in economic problems and partner unemployment than White women. Women with higher education reported more work problems, but fewer economic problems and less partner unemployment. Family health-related events decreased until women reached 55–60 years, then increased again. White women reported more illnesses and accidents among loved ones; Black women reported more caregiving and bereavement; and Hispanic women reported greater declines in early midlife and larger increases in later midlife on accidents and deaths. Overall relationship problems, legal/police problems, and violence to self or family declined as women aged. Black women experienced less decline in events in these categories as they aged and White women were more likely than Hispanic, Chinese, and Japanese women to report these events. Hispanic women reported greater increases in violent events and family legal/police problems in later midlife [9]. These results suggest the importance of understanding the trajectories of stressful life events during midlife and the multiple factors associated with them.

The purpose of the group-based trajectory models (GBTMs) presented here is to identify the trajectories of undesirable and desirable life events among midlife women over a decade of their lives using a sample from the SMWHS. Predictors include socio-economic factors (income, education, race/ethnicity, employment), demographic characteristics (age, marital/partner status, being a parent), and reproductive aging stages that may influence those patterns.

## Methods

### Study design and population

This investigation was part of a larger study, the Seattle Midlife Women’s Health Study (SMWHS), an observational, longitudinal study of approximately 23 years, from 1990 to 2013. Women entered the study between 1990 and early 1992, when most were in the early stages of the menopausal transition or not yet in the transition. All households within census tracts with a wide income range and mixed race/ethnicity were contacted and screened by telephone for interested and eligible women. Women who were eligible were between 33 and 55 years of age, had at least one menstrual period within the last year, had a uterus and at least one ovary, were not pregnant, and could read and understand English. Women were ineligible if they had a bilateral oophorectomy, a total hysterectomy with both ovaries removed, became pregnant, could not read or write English, or were in the postmenopausal transition stage. Out of 11,222 telephone contacts, 820 women were eligible, and 508 women entered the study [10]. Women completed an initial in-person interview administered by a trained registered nurse interviewer. A subset of the 508 women kept a health diary. All women were mailed a yearly health questionnaire and kept a menstrual calendar.

### Sample size

Of the 508 women who entered the study, only 380 are included in these analyses. Participants of the current study were those women who provided at least one and up to four Life Event Scale (LES) questionnaires beginning in 1990 (Occasion/Time point 1) and who were in either the late reproductive (LR), early transition (ET), late transition (LT) or post-menopause (PM) stage sometime during the course of the study.

The Life Event Scale (LES) was administered on four time points: Baseline (1990), Year 2 (1993), Year 7 (1997), and Year 10 (2000). Attrition rates for the initial LES for baseline included 67 women who were unable to be contacted, 5 women who became ineligible, and 64 who left the study for personal reasons leaving a total of 380 women. For year 2, thirty-six women were unable to be contacted, a total of 18 women became ineligible, and 32 left for personal reasons resulting in 233 women. During year seven, eighteen women were unable to be contacted, ten became ineligible, and 15 women left for personal reasons generating 220 participants. For year 10, nineteen women were unable to be contacted, 138 became ineligible, 34 left for personal reasons leaving 191 women.

### Measures

The measures used in this analysis included the Life Event Scale (LES) and menstrual calendars to determine menopausal transition stage. Socio-economic factors such as income, years of education, race/ethnicity, employment, and demographic variables including age, marital status, being a parent, and menopausal transition stage were used to identify the women’s baseline characteristics (see Table 1) and to identify predictors of the four GBTMs.

**Table 1:** Socio-economic factors and demographic variables of Seattle Midlife Women’s Health Study participants providing data for the Life Events Scale over four time points

Characteristic	Time point 1 N = 380	Time point 2 N = 233	Time point 3 N = 220	Time point 4 N = 191
	Mean (SD)	Mean (SD)	Mean (SD)	Mean (SD)
Socio-economic factors				
Gross Family Income	35,820 (15,400)	54,000 (only 1)	41,000 (15,000)	43,600 (14,400)
Years of Education	15.5 (2.8)	15.9 (2.6)	15.9 (2.5)	16.0 (2.5)
Race/Ethnicity African American Asian/Pacific Islander White Latina Mixed/Native Amer.	11.6% 8.9% 75.8% 1.1% 2.6%	8.2% 8.6% 80.7% 1.3% 1.3%	6.8% 8.6% 84.1% 0.5% 0	6.8% 8.9% 84% 0 0
Currently Employed	85.3%	95.7%	88.2%	89.1%
Demographic variables				
Age	41.6 (4.6)	43.8 (4.6)	47.1 (4.3)	49.8 (4.2)
Marital Status Never married/partnered Married/partnered Divorced/separated Widowed	5.8% 70.8% 21.8% 1.6%	6.0% 65.2% 27.9% 0.9%	5.5% 67.7% 25.0% 1.8%	3.7% 65.4% 29.8% 1.0%
If a parent? Yes No	74.7% 25.3%	67.4% 32.6%	70.5% 29.5%	67.5% 32.5%
Menopausal Transition Stage (MTS), % (N) Late Reproduction (LR) Early Transition (ET) Late Transition (LT) Post Menopause (PM)	70.3% (N = 142) 22.3% (45) 5.4% (11) 2.0% (4)	58.1% (100) 33.1% (57) 4.7% (8) 4.1% (7)	43.1% (66) 37.3% (57) 13.1% (20) 6.5% (10)	37.6% (47) 24.8% (31) 21.6% (27) 16.0% (20)

#### The life event scale

The Life Events Scale (LES) was adapted for use with midlife women by the investigators of the SMWHS from Norbeck’s Life Event Questionnaire [6]. An enumeration of each item is given in a previously published appendix [8]. The LES is a 77-item, self-rated scale that assesses whether or not a stressful event happened over the past year and how stressful the event was. The LES was given four times during the course of the SMWHS: Baseline, Year 2, Year 7, Year 10. The LES has the same 9 sections or categories as the LEQ: Health, Work, Residence, Love & Marriage, Family & Close Friends, Parenting, Personal & Social, Financial, and Crime & Legal matters.

The LES is different from the LEQ in the wording of the questions seen in the categories of Work; Family & Close Friends; and, Personal & Social. The following items were changed to reflect relevance to midlife. In the Work category, one item, “job changed,” was added. For Family & Close Friends, “death of parents” and “birth of a grandchild” were added; and, “acquired or lost a pet” was omitted. The questions were adapted in item 5a (see Appendix [8]) to include “grandchild” and in item 5 g to include “other family members (than a parent).” In the Personal & Social category, one item was added, “lost a friend for other reasons.” In summary, after the adjustment for midlife women, both the LES and LEQ totaled 77 items.

Women were asked whether or not a life event occurred during the last year (yes/no); to evaluate if the event was desirable, neutral, or undesirable; and, to rate the impact of the event as (1) no effect, (2) small effect, (3) moderate effect, or (4) great effect. For this investigation, mean impact total scores, mean impact undesirable scores, and mean impact desirable scores were reported for all nine sections of the LES over four separate occasions spanning 10 years.

#### Menopausal transition stages

Menopausal transition stages were labeled according to the stages of reproductive aging [11]: Late reproductive stage (LR), early menopausal transition stage (ET), late menopausal transition stage (LT), or post-menopause (PM), and match those labels from the Stages of Reproductive Aging Workshop (STRAW) [12] and STRAW + 10 [13–15]. The data were obtained by menstrual calendars and coded as LR, ET, LT, or PM based on criteria developed by The Seattle Midlife Women’s Health Study (SMWHS) [11] and validated by Harlow and colleagues [15, 16]. The late reproductive (LR) stage includes the time in midlife before the onset of persistent cycle irregularity when cycles are regular; Early transition (ET) stage is defined as persistent irregularity of more than six days of absolute difference between the start of any two consecutive menstrual cycles during the year with no skipped periods; Late transition (LT) is defined as persistent skipping of one or more menstrual periods. A skipped period was defined as 60 or more consecutive days of amenorrhea during the calendar year [16]. Persistence indicated that the irregular cycle or skipped period took place one or more times in the ensuing 12 months. The final menstrual period (FMP) was identified retrospectively after one year of amenorrhea. The first day of the FMP was used to determine age of onset of the FMP. Early post-menopause (PM) was the time frame within five years after the FMP.

#### Socio-economic factors and demographic variables

Income from all sources was measured as gross family monthly pay in dollars. Education was measured in years. Ethnicity was self-reported as Latina or not, and race was reported as African American, Asian/Pacific Islander, Mixed/Native American, or White. Current employment (full time or part time) was assessed using employed or not employed. Age was also measured in years. Marital status was self-reported as never married/never partnered, married/partnered, divorced/separated, or widowed. Parental status was determined by asking women whether or not they were parents, including parenting adopted or foster-children. See Table 1.

### Data analysis

Descriptive statistics (mean, standard deviation) were used to describe the individual items of the Life Event Scale (LES, see appendix [8]), scale total scores, impact scores of the LES, and socio-economic/demographic variables by time point when the questionnaires were administered. Descriptive statistics were performed by SPSS v23.

#### Group-based trajectory model (GBTM) analysis

GBTM [17] analysis distinguishes subgroups of women who follow different patterns of change over time obtaining a more complete representation of the data. GBTM allows the researcher to better understand the intra- and inter- variability of growth patterns over time in comparison to using average estimates that may oversimplify the intra- and inter- variability of real life [18]. For women in the SMWHS, GBTM used finite mixtures of probability distributions to identify clusters of individual trajectories. Maximum likelihood was used to estimate the model parameters. The censored normal distribution was used since it was designed for the analysis of repeated measures of continuous outcomes bounded by scale minimums and maximums [17]. Of interest were the outcome trajectories for SLEs over time.

The analysis was conducted using the following steps. Descriptive data for each of the four models (total scores for undesirable events, total impact scores for undesirable events, total scores for desirable events, and total impact scores for desirable events) were calculated using mean, standard deviation, median, and number of women for each of the four time points. The data were reviewed for normalcy and were transformed. The square root transformation provided a more normal approximation to the data. The total scores models used quadratic terms. The GBTM analyses identified two to six trajectories per model. See Supplementary Information for the following tables: Table 2. Model parameters of total number of undersirable events, Table 3. Model parameters of total impact scores for undesirable events, Table 4. Model parameters of total number of desirable events, and Table 5. Model parameters of total impact scores for desirable events. The number of trajectories was determined based on several factors. These factors were the relative fit information criteria, such as the Bayesian Information Criteria (BIC) for choosing models that had lower absolute values that indicated better fit models; parsimony of the trajectories; theoretical interpretation; and statistical significance of an appropriate number of trajectories. Analyses were conducted with STATA v14.1 software [19] using TRAJ [20].

#### Missing data

The total value of the responses for each category of the LES was divided by the number of questions answered. For example, if a participant answered two out of seven parts of the Health category of the LES and rated their impact as a 3 and a 4, the total (3 + 4 = 7) would be divided by 2, so 3.5 would be the average impact score for the Health category of the LES for that year.

#### Multinomial logistic regression

Multinomial logistic regression analysis was used to identify predictors of each of the four GBTMs using baseline data. Predictors in the model included socio-economic factors of gross family income, education, race/ethnicity, employment, and demographic characteristics of age, marital status, being a parent, and menopausal transition stage (MTS). MTS was divided into LR, ET, and LT/PM. Since there were four to five women in the PM stage, the PM stage was added to the LT stage to make that group more robust.

## Results

### Descriptive statistics of the sample

When the women started the study, they were on average 42 years, well-educated (16 years of education), and earning a gross family income of $35,820 (SD $15, 400). Most of the women were employed (85%) and married (71%). Women identified themselves as African American (12%), Asian/Pacific Islander (9%), White (76%), Latina (1%), and Mixed/Native American (3%). Approximately 75% of the women were parents (See Table 1).

The mean age of the women increased at each testing occasion (*F* = 169, *df* = 3, *p* < .001) from 42 years to 50 years, on average, as expected for a longitudinal study. The number of years of education of the women remained the same over time. The participants who remained in the study earned higher incomes over time, perhaps suggesting that those women with lower incomes tended to leave the study (*F* = 18.602, *df* = 2, *p* < .001). At least 86% of the women were employed as indicated by a higher percentage reporting employment on each occasion and the possibility that those who were not employed were more likely to leave the study (Pearson’s Chi square = 18.864, *df =* 3, *p* < .001). The diversity of the sample also changed over time (Chi square = 18.907, *df* = 9, *p* = .026) resulting in a higher proportion of White women remaining enrolled in the study with fewer African American, Latina, and Mixed/Native American women completing the study. There were no significant differences between occasions for marital status (Pearson’s Chi square = 7.221, *df* = 9, *p* = .614), although there was a slight decrease in the percentage of married women, suggesting that these women divorced or dropped out of the study; there was a slight increase in the percentage of divorced women over the course of the study. There was a slight decrease, although not statistically significant, of the number of parents who remained in the study (Pearson’s Chi square = 5.161, *df* = 3, *p* = .160). As anticipated, there were significant differences among the women in the different menopausal transition stages across all occasions (Chi square = 81.440, *df* = 9, *p* < .001) with women progressing from the late reproductive (LR) to early (ET) and late (LT) transition and then to post-menopause (PM).

### Group-based trajectory models (GBTM)

**Fig. 1:**
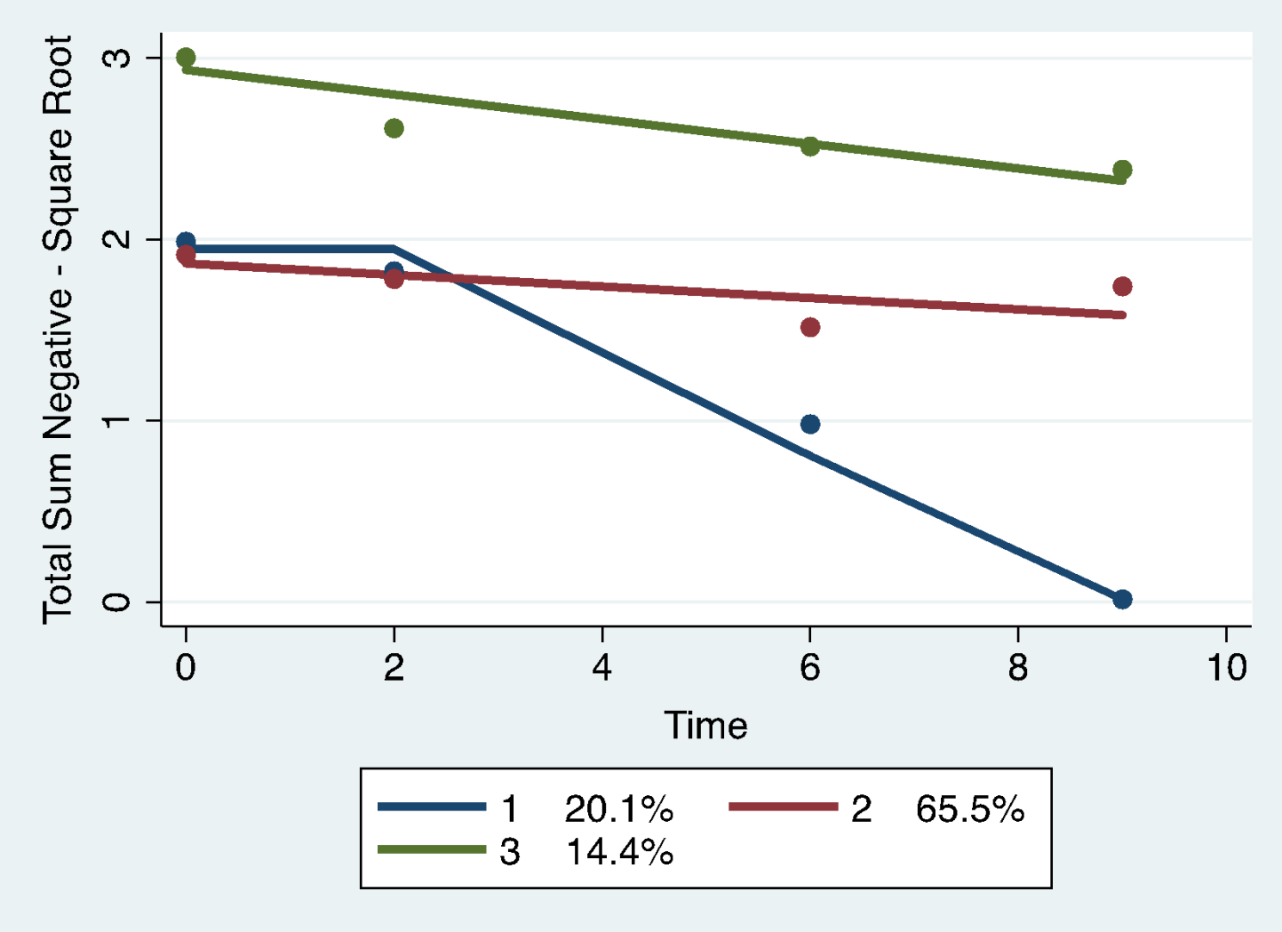
Total number of undesirable life events: group-based trajectory models. Trajectory 1. Blue line. Medium sharp decreasers, N = 76 women, 20.1% (SE 3.10). Trajectory 2. Red line. Medium high sustainers, N = 249 women, 65.5% (SE 5.49). Trajectory 3. Green line. High sustainers, N = 55 women, 14.4% (SE 4.89)

**Fig. 2:**
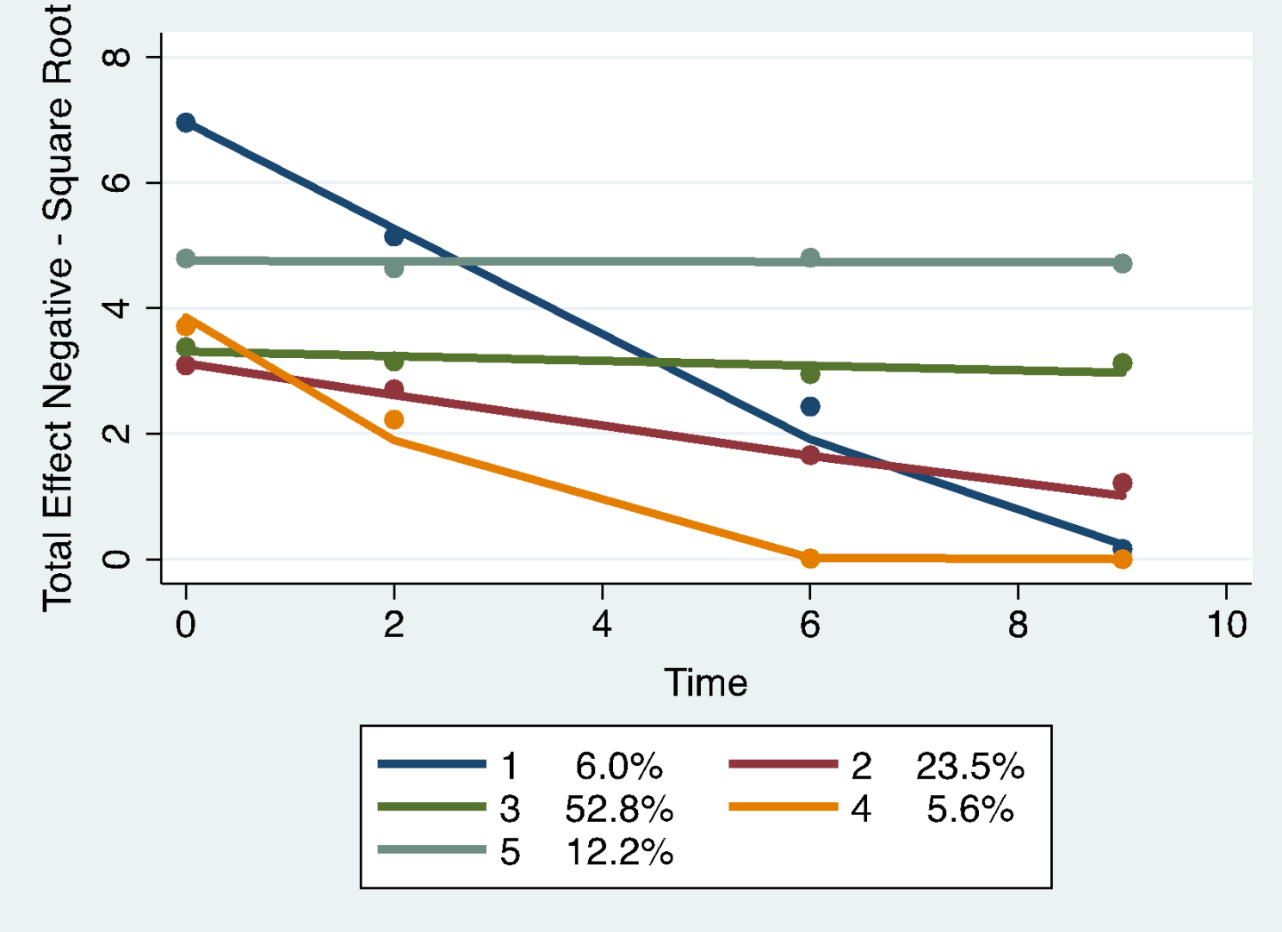
Total impact scores for undesirable life events: group-based trajectory models. Trajectory 1. Dark blue. Sharp decreasers, N = 23 women, 6.0% (SE 1.91). Trajectory 2. Red. Low levelers, N = 89 women, 23.5% (SE 7.13). Trajectory 3. Green. Medium low sustainers, N = 200 women, 52.5% (SE 7.39). Trajectory 4. Orange. Medium sharp decreasers, N = 21 women, 5.6% (SE 1.79). Trajectory 5. Grey. Medium sustainers, N = 47 women, 12.2 % (SE 4.13)

**Fig. 3:**
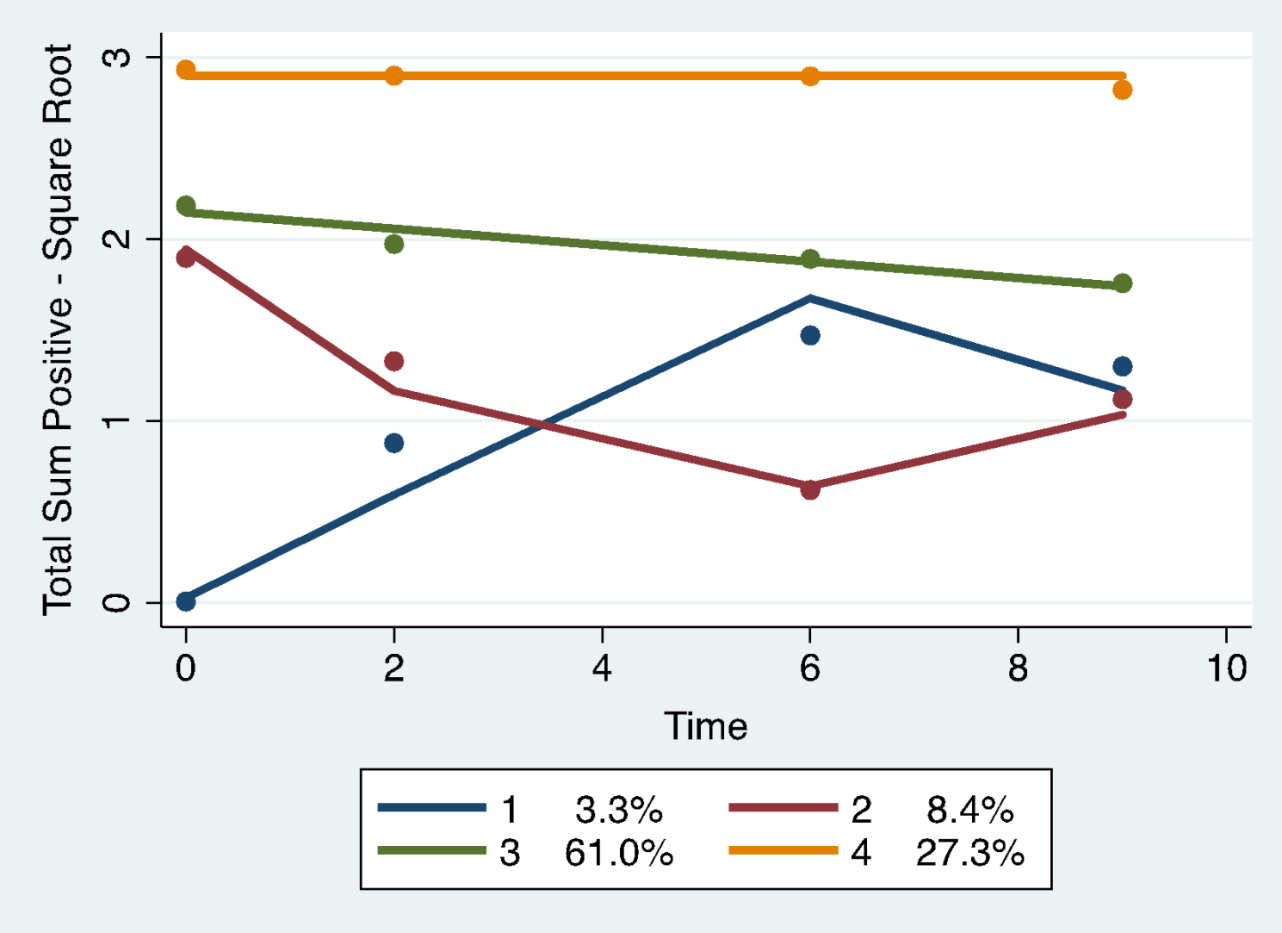
Total number of desirable life events: group-based trajectory models. Trajectory 1. Dark blue. Low to medium increasers, N = 12 women, 3.3% (SE 0.93). Trajectory 2. Red. Medium to low decreasers, N = 32 women, 8.4% (SE 4.63). Trajectory 3. Green. Moderately high sustainers, N = 232 women, 61% (SE 5.03). Trajectory 4. Orange. High sustainers, N = 104 women, 27.3% (SE 4.19)

**Fig. 4:**
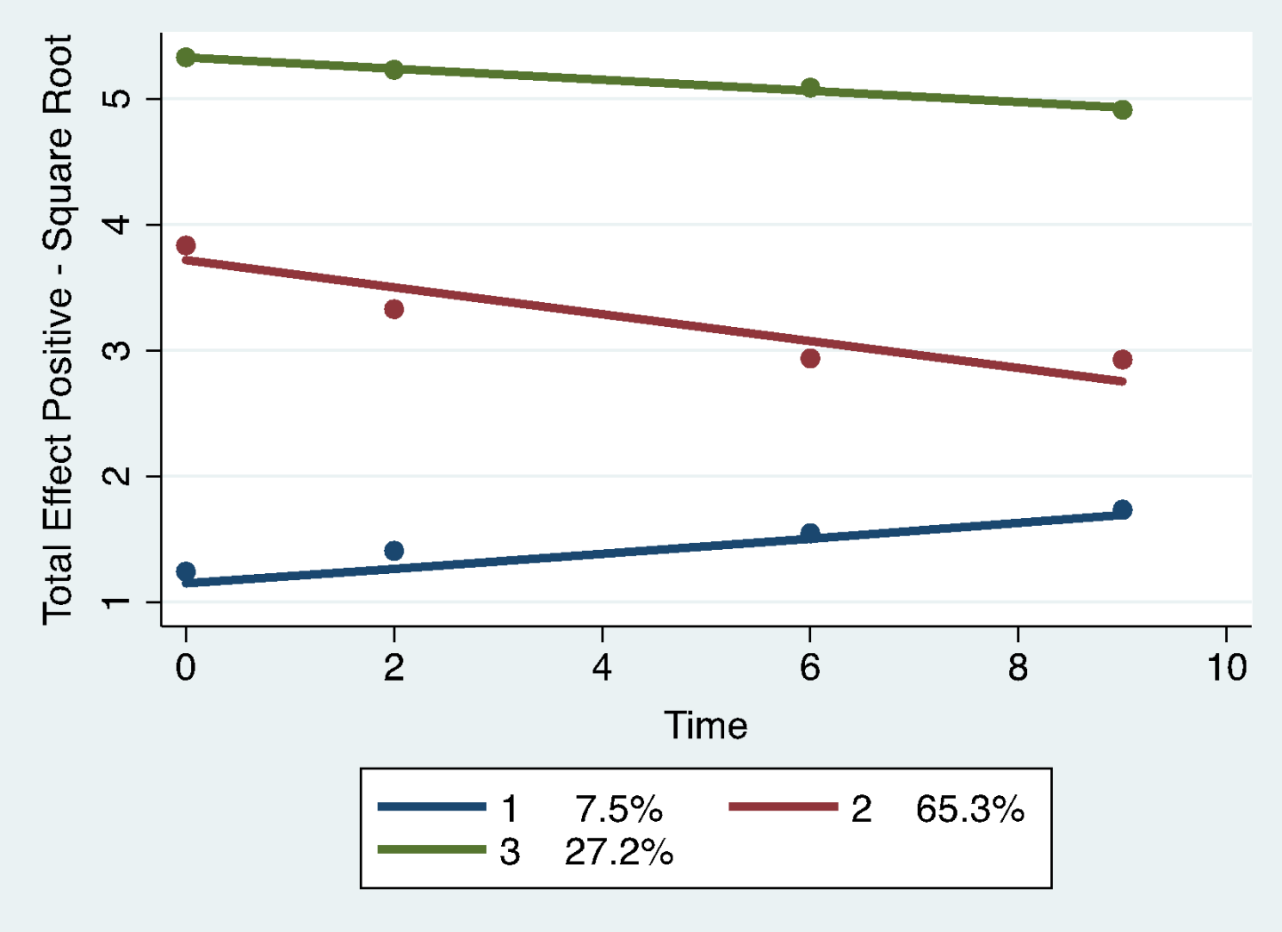
Total impact scores for desirable life events: group-based trajectory models. Trajectory 1. Dark blue. Low sustainers, N = 29 women, 7.5% membership (SE 2.38). Trajectory 2. Red. Medium sustainers, N = 248 women, 65.3% (SE 4.41). Trajectory 3. Green. High sustainers, N = 103 women, 27.2% (SE 4.43)

Trajectory analysis revealed four different group-based trajectory models. The models included: total number of undesirable events, total impact scores for undesirable events, total scores for desirable events, and total impact scores for desirable events. See Figs. 1, 2, 3 and 4; Tables 2, 3, 4 and 5.

#### Total number of undesirable life events

were defined as the sum of the number of items answered as undesirable in all categories. A three-trajectory model was selected due to the best fit index of the Bayesian Information Criterion (BIC= -1303.23, N = 458 observations). See Fig. 1; Table 2. The first trajectory started moderately high and had a sharp decrease (medium decreasers) from year 2 to year 10 and included 20.1% (SE 3.10, N = 76 women) of the participants. Trajectory 2 (65.5% membership; SE 5.49, N = 249 women) started moderately high and declined slightly through year 10 (medium high sustainers). Trajectory 3 started high and had a moderate decline over time (high sustainers) and included 14.4% of the participants (SE 4.89, N = 55 women).

#### Total impact scores for undesirable life events

were defined as the sum of all the impact scores of an undesirable event, which was evaluated as none (1), some (2), moderate (3) or great (4). The best model as indicated by the Bayesian Inclusion Criterion (BIC = -1907.80, N = 458 observations) included 5 trajectories. See Fig. 2; Table 3. Trajectory 1 (6% membership; SE 1.91, N = 23 women) started out high at baseline and sharply decreased to very low at year 10 (sharp decreasers). Trajectory 2 (23.5% membership; SE 7.13, N = 89 women) started moderately low and decreased to low from year 7 to year 10 (low levelers). Trajectory 3 (52.5% membership; SE 7.39, N = 200 women) was slightly less than medium at baseline and remained slightly less than medium throughout the 10 years of the study (medium low sustainers). Trajectory 4 (5.6% membership; SE 1.79, N = 21 women) started at a medium level at baseline sharply decreasing to very low from year 7 to year 10 (medium sharp decreasers). Trajectory 5 (12.2% membership; SE 4.13, N = 47 women) started out as medium and remained at a medium level throughout the 10 years of the study (medium sustainers).

#### Total number of desirable life events

were defined as the sum of the number of items answered as desirable. The censored normal model [17] identified four trajectories of women over a 10-year time period. The Bayesian Information Criterion (BIC) was relied upon for model selection (BIC= -1238.37, N = 458 observations). See Fig. 3; Table 4. Trajectory 1 (3.3% membership; SE 0.93, N = 12 women) started very low, increased to moderate until year seven and moderately declined to year 10 (low to medium increasers). Trajectory 2 (8.4% membership; SE 4.63, N = 32 women) started moderately high, decreasing to low at year 7 with a moderate increase to year 10 remaining at a lower level (medium to low decreasers). Trajectory 3 (the highest group membership at 61%; SE 5.03, N = 232 women) was moderately high very slightly decreasing to year 10 (moderately high sustainers). Trajectory 4 (27.3% membership; SE 4.19, N = 104 women) started very high and remained high throughout the 10 years of the study (high sustainers).

#### Total impact scores for desirable life events

were defined as the evaluation of a desirable event as none (1), some (2), moderate (3) or great (4). The best model as indicated by the Bayesian Inclusion Criterion (BIC = -1893.24, N = 458 observations) included three trajectories. See Fig. 4; Table 5. Trajectory 1 (7.5% membership; SE 2.38, N = 29 women) was very low at baseline, increased slightly, and remained low at year 10 (low sustainers). Trajectory 2 (65.3% membership; SE 4.41, N = 248 women) was medium high at baseline decreasing to medium at year 10 (medium sustainers). Trajectory 3 (27.2% membership; SE 4.43, N = 103 women) was very high at baseline and decreased slightly remaining high at year 10 (high sustainers).

### Multinomial logistic regression predictors

Neither the socio-economic nor the demographic variables were significant predictors of trajectory membership in any of the four models. The MTS consisting of LR, ET, and LT/PM groups were not significant predictors in any of the four growth curve models.

## Discussion

The SMWHS is one of the few studies using group-based trajectory modeling to identify subgroups of women with similar longitudinal exposures to SLEs using predictors of socio-economic factors, demographic characteristics, and menopausal transition stage. The purpose of the analyses presented here was to create a GBTM to identify patterns of undesirable and desirable life events for midlife women over a decade. Four different trajectory models were identified using the Life Event Stress (LES) scale over four time points of the SMWHS spanning 10 years. The models were (1) Total Number of Undesirable SLEs, (2) Total Impact Scores for Undesirable SLEs, (3) Total Number of Desirable SLEs, and (4) Total Impact Scores for Desirable SLEs.

The first analysis of the GBTM, Total Number of Undesirable Events, identified three trajectories. Approximately 86% of women had medium high exposure to undesirable SLEs with a slight decrease (65.5%) or a sharp decrease (20.1%) over 10 years. Some women (14.4%) had high exposure with a very slight decrease over time. All three trajectories of the first analysis indicated that most of these women have had a large amount of sustained stress for the past 10 years although all trajectories decreased over time. In midlife, women often have many stressful life event challenges that co-occur. These co-occurring challenges may include divorce, loss of income, and loss of health insurance, for example [8]. Chronic SLE sustained stress ratings are concerning because they may contribute to allostatic load. Allostasis is a state where the body maintains homeostasis and adaptation with the use of biological mediators (neurotransmitters, immune system messengers, and the hormones cortisol and epinephrine) that are activated by stressors to the body and mind. Problems occur when the mediators are (1) not activated by stressors or (2) not turned off after they have been activated. Over time, continuation of constant stressors may result in allostatic overload. Allostatic load indicates that chronic stress may lead to a change in personal behaviors or lifestyle that would result from overuse or “wear and tear” [21]. Allostatic *over*load refers to the pathophysiology that occurs when the negative experiences accumulate over time and are repeated over and over again without anything to offset the continual stress. This type of pathophysiology may manifest itself in cardiac problems, diabetes mellitus, arthritis, hypertension, obesity, stroke [22], and sleep loss [23, 24]. Life events have been associated with an increased risk for health problems. One German study [25] using a cross-sectional design found that a summation of SLEs was associated with an increased risk of cardiovascular risk factors and myocardial infarction and that with every additional SLE, the odds increased for obesity (50% for women), for diabetes (52% for women), and for myocardial infarction (110% for women).

The second analysis used a summation of impact scores to understand the influence or assessment of the undesirable life events. The Total Impact Scores for Undesirable Events revealed five trajectories. The majority of women (approximately 64% from trajectories 3 and 5) had moderate, sustained impact ratings while approximately 35% had impact ratings that decreased over time (trajectories 1, 2, and 4). Some of the women may have had new diagnoses of personal health problems or diagnoses of other family members, for example, that continued to impact their lives. Evidence indicates that the impact for undesirable SLEs was the greatest in the categories of family/friends, personal/social, work, and health [8]. Women whose ratings decreased over time may not have had life events that had a strong impact on their lives, or the women may have acquired wisdom or other coping mechanisms that enabled them to rate the life events that they experienced lower, what Haehner et al. [26] refer to as cognitive coping where a person finds an explanation for the event making the event less extraordinary over time. Therefore, their appraisals may have changed or moderated over the years.

What may help to offset chronic repeated stress are positive or desirable events.

The third analysis, Total Number of Desirable events, produced four trajectories. Most women (approximately 88%) were high and medium high sustainers, meaning their trajectory was high over ten years, which may help to balance or offset the high ratings of undesirable life events. For example, a wedding or buying a house may be a desirable, although stressful, event that would counter a divorce or death of a loved one. One study from the Netherlands [27] indicated that positive affect, which may occur after desirable SLEs, was associated with a more favorable allostatic load profile, especially in women. There were two subgroups of women who had very low numbers of desirable events; one trajectory (8.4%) declined over the first 3 occasions slightly increasing toward the end. This trend may suggest that there were a low number of women who experienced desirable life events or that there were a low number of desirable events. Evidence with younger participants (average age of 22 years) suggests that the response after a SLE may decrease over time as the past is re-interpreted in a less stressful way because explanations of the SLE are found [26]. Midlife women may also re-interpret their responses in the same manner.

The fourth analysis, Total Impact Scores for Desirable Events, identified three trajectories. Most ratings started high (approximately 27%) or medium high (65%) and slightly decreased over time. Group probability was approximately 8% for women whose ratings were very low and slightly increased over time. Women’s ratings of the desirable events impact decreased over time which may suggest that desirable life events had less effect on them, or another possibility is that the number of desirable SLEs decreased over time. Psychological and social changes in midlife are often associated with positive changes [28]. Some examples of positive changes are improved emotional regulation [29], mastery [30], and increased wisdom [31]. These positive changes may have had an effect on how these midlife women rated their desirable SLEs.

Socio-economic factors (income, education, race/ethnicity, and employment) and demographic characteristics (age, marital status, being a parent), and menopausal transition stage may influence the trajectory of stress over time. Most of the women in this sample of the SMWHS were well-educated, employed, married, and white, perhaps why the current study did not identify any of these predictors as significant. Nonetheless, inequities lie in these socio-economic factors and demographic characteristics and these inequities create stress. Consider, for instance, single midlife women who have lower income who may not have the ability to pay for healthcare for their family, or a midlife woman of color who may be discriminated against in applying for a higher position in her workplace due to her gender or ethnicity.

This study has limitations. The current study’s sample size was smaller (N = 380 at baseline) compared to the SWAN studies where in one study [3], the sample size was over 3000 women at baseline. The sample in the current study included mostly white women, although the investigators tried to recruit more women of color. Another limitation is the normal attrition that occurs during longitudinal studies. One of the strengths of this study is that chronic life event stress was investigated in midlife women repeatedly over a decade.

Life event stress may reflect the impact of the chronic activation (stress arousal) of the hypothalamic-pituitary-adrenal (HPA) axis. Future investigations may consider how, and if, desirable or positive life events buffer the effects of negative or undesirable stressful life events on HPA axis responses, such as cortisol levels.

## Conclusion

The current study advances the literature on longitudinal trajectories of life events of women in midlife who are also going through the menopausal transition. The patterns of GBTM of subgroups of women with similar exposure to life events may be useful to identify vulnerable populations, such as women who have high levels of undesirable sustained stress. This information may provide evidence to health professionals to help personalize healthcare interventions for each individual woman to alleviate higher levels of stress and reduce the risk of allostatic load.

### Electronic supplementary material

Below is the link to the electronic supplementary material.

**Table 2**. Model parameters of total number of undesirable events. **Table 3**. Model parameters of total impact of undesirable events. **Table 4**. Model parameters of total scores for desirable events. **Table 5**. Model parameters of impact scores for desirable events.

Supplementary Material 2

## Data Availability

Data analyses are still in progress. In the future, the data base can be available for other investigators.
