# Supplementary material for: Stressful life events during the perimenopause: longitudinal observations from the seattle midlife women’s health study
Source: Womens Midlife Health. 2023 Sep 5;9:6. doi: 10.1186/s40695-023-00089-y (PMC10478480; doi:10.1186/s40695-023-00089-y)
Supplement: Supplementary file 1 — Table 2. Model parameters of total number of undesirable events. Table 3. Model parameters of total impact of undesirable events. Table 4. Model parameters of total scores for desirable events. Table 5. Model parameters of impact scores for desirable events. [file 40695_2023_89_MOESM1_ESM.docx]

**Table 2. Model parameters of total number of undesirable events.**

**Descriptive data of total scores for undesirable events**

| LES time point/occasion | Mean | SD | Median | N |
| --- | --- | --- | --- | --- |
| 1 | 5.02 | 3.82 | 4 | 380 |
| 2 | 3.98 | 2.71 | 3 | 233 |
| 3 | 3.18 | 2.97 | 2 | 220 |
| 4 | 3.13 | 3.01 | 3 | 191 |

Maximum Likelihood Estimates

Model: Censored Normal

**Four classes: total scores for undesirable events**

| Group | Parameter | Standard Estimate | Standard Error | T for HO:  Parameter=0 | Prob > \|T\| |
| --- | --- | --- | --- | --- | --- |
| 1 | Intercept  Linear | 1.96  -0.16 | 0.10  0.03 | 19.08  -6.25 | 0.00  0.00 |
| 2 | Intercept  Linear | 1.94  -0.01 | 0.07  0.01 | 27.18  -0.85 | 0.00  0.39 |
| 3 | Intercept  Linear | 2.38  -0.60 | 0.36  0.11 | 6.71  -5.33 | 0.00  0.00 |
| 4 | Intercept  Linear | 3.21  -0.10 | 0.22  0.04 | 14.43  -2.68 | 0.00  0.01 |
|  | Sigma | 0.71 | 0.02 | 33.420 | 0.00 |
| Group Membership |  |  |  |  |  |
| 1 | % | 28.94 | 6.29 | 4.60 | 0.00 |
| 2 | % | 55.94 | 6.70 | 8.35 | 0.00 |
| 3 | % | 6.07 | 1.79 | 3.40 | 0.00 |
| 4 | % | 9.05 | 3.43 | 2.64 | 0.01 |

BIC=Bayesian information criterion, AIC=Akaike information criterion, L=Log likelihood maximum criterion

BIC = **-1304.72** (N=458) AIC = -1279.96 L= -1267.96

**Three classes: total scores for undesirable events**

| Group | Parameter | | Standard Estimate | | Standard Error | | T for HO:  Parameter=0 | Prob > \|T\| |
| --- | --- | --- | --- | --- | --- | --- | --- | --- |
| 1 | Intercept  Linear  Quadratic | | 1.95  0.10  -0.05 | | 0.12  0.10  0.01 | | 15.77  0.99  -3.66 | 0.00  0.32  0.00 |
| 2 | Intercept  Linear | | 1.87  -0.03 | | 0.06  0.01 | | 30.18  -3.22 | 0.00  0.00 |
| 3 | Intercept  Linear | | 2.93  -0.07 | | 0.20  0.03 | | 14.53  -2.59 | 0.00  0.01 |
|  | sigma | | 0.72 | | 0.02 | | 34.50 | 0.00 |
| Group Membership | |  | |  | |  |  |  |
| 1 | % | | 20.10 | | 3.10 | | 6.48 | 0.00 |
| 2 | % | | 65.48 | | 5.49 | | 11.92 | 0.00 |
| 3 | % | | 14.42 | | 4.89 | | 2.95 | 0.00 |

BIC = **-1303.23*** (N=458) AIC = -1282.59 L = -1272.59

**Two classes: total scores of undesirable events**

| Group | Parameter | | Standard Estimate | | Standard Error | T for HO:  Parameter=0 | | Prob > \|T\| |
| --- | --- | --- | --- | --- | --- | --- | --- | --- |
| 1 | Intercept  Linear | | 2.04  -0.24 | | 0.13  0.03 | 16.05  -7.82 | | 0.00  0.00 |
| 2 | Intercept  Linear | | 2.09  -0.04 | | 0.05  0.01 | 38.98  -3.31 | | 0.00  0.00 |
|  | sigma | | 0.79 | | 0.02 | 39.99 | | 0.00 |
| Group Membership |  |  | |  | |  |  | |
| 1 | % | | 27.41 | | 4.79238 | 5.718 | | 0.00 |
| 2 | % | | 72.60 | | 4.79238 | 15.148 | | 0.00 |

BIC = **-1305.64** (N=458) AIC = -1293.26 L= -1287.26

**Five classes: total scores of undesirable events**

| Group | Parameter | | Standard Estimate | | Standard Error | T for HO:  Parameter=0 | Prob > \|T\| |
| --- | --- | --- | --- | --- | --- | --- | --- |
| 1 | Intercept  Linear | | 1.84  -0.15 | | 0.10  0.03 | 17.86  -5.83 | 0.00  0.00 |
| 2 | Intercept  Linear | | 1.91  -0.02 | | 0.08  0.01 | 24.67  -1.14 | 0.00  0.26 |
| 3 | Intercept  Linear | | 2.65  -0.02 | | 0.21  0.03 | 12.43  -0.76 | 0.00  0.45 |
| 4 | Intercept  Linear | | 2.13  -0.55 | | 0.23  0.09 | 9.12  -6.03 | 0.00  0.00 |
| 5 | Intercept  Linear | | 3.70  -0.45 | | 0.23  0.07 | 16.13  -6.91 | 0.00  0.00 |
|  | sigma | | 0.67 | | 0.02 | 31.95 | 0.00 |
| Group Membership |  |  | |  | |  |  |
| 1 | % | | 23.68 | | 6.04 | 3.92 | 0.00 |
| 2 | % | | 53.36 | | 7.23 | 7.38 | 0.00 |
| 3 | % | | 11.45 | | 5.67 | 2.02 | 0.04 |
| 4 | % | | 5.74 | | 1.69 | 3.39 | 0.00 |
| 5 | % | | 5.76 | | 1.99 | 2.90 | 0.00 |

BIC = **-1305.20** (N=458) AIC = -1274.25 L= -1259.25

**Three-class model is best fit for total number of undesirable events determined by the lowest [absolute] BIC.*

**Table 3. Model parameters of total impact of undesirable events.**

**Descriptive data for total impact of undesirable events**

| LES time point/occasion | Mean | SD | Median | N |
| --- | --- | --- | --- | --- |
| 1 | 16.24 | 13.69 | 12 | 380 |
| 2 | 12.25 | 9.53 | 9 | 233 |
| 3 | 9.99 | 10.12 | 7 | 220 |
| 4 | 9.87 | 10.77 | 8 | 191 |

Maximum Likelihood Estimates

Model: Censored Normal

**Four classes: total impact of undesirable events**

| Group | Parameter | Standard Estimate | | Standard Error | | T for HO:  Parameter=0 | | Prob > \|T\| |
| --- | --- | --- | --- | --- | --- | --- | --- | --- |
| 1 | Intercept  Linear | 4.31  -1.11 | | 0.72  0.24 | | 5.98  -4.61 | | 0.00  0.00 |
| 2 | Intercept  Linear | 3.41  -0.03 | | 0.15  0.03 | | 22.36  -0.87 | | 0.00  0.39 |
| 3 | Intercept  Linear | 3.34  -0.27 | | 0.19  0.05 | | 17.99  -5.05 | | 0.00  0.00 |
| 4 | Intercept  Linear | 5.93  -0.15 | | 0.40  0.06 | | 14.97  -2.44 | | 0.00  0.02 |
|  | sigma | 1.33 | | 0.04 | | 33.91 | | 0.00 |
| Group Membership |  |  | |  | |  | |  |
| 1 | % | | 5.84 | | 1.84 | | 3.18 | 0.00 |
| 2 | % | | 52.42 | | 8.93 | | 5.87 | 0.00 |
| 3 | % | | 32.02 | | 8.93 | | 3.59 | 0.00 |
| 4 | % | | 9.72 | | 3.29 | | 2.95 | 0.00 |

BIC=Bayesian information criterion, AIC=Akaike information criterion, L=Log likelihood maximum criterion

BIC = **-1911.36** (N=458) AIC = -1886.6 L= -1874.60

**Five classes: total impact of undesirable events**

| Group | Parameter | Standard Estimate | | Standard Error | | T for HO:  Parameter=0 | | Prob > \|T\| |
| --- | --- | --- | --- | --- | --- | --- | --- | --- |
| 1 | Intercept  Linear | | 6.97  -0.85 | 0.42  0.13 | | 16.61  -6.82 | | 0.00  0.00 |
| 2 | Intercept  Linear | | 3.12  -0.26 | 0.20  0.05 | | 15.81  -4.98 | | 0.00  0.00 |
| 3 | Intercept  Linear | | 3.31  -0.04 | 0.14  0.03 | | 24.37  -1.51 | | 0.00  0.13 |
| 4 | Intercept  Linear | | 3.86  -1.00 | 0.50  0.19 | | 7.69  -5.21 | | 0.00  0.00 |
| 5 | Intercept  Linear | | 4.76  -0.00 | 0.30  0.05 | | 15.90  -0.06 | | 0.00  0.96 |
|  | sigma | | 1.25 | 0.04 | | 32.32 | | 0.00 |
| Group Membership |  | |  |  | |  | |  |
| 1 | % | | 5.95 | | 1.91 | | 3.12 | 0.00 |
| 2 | % | | 23.49 | | 7.15 | | 3.28 | 0.00 |
| 3 | % | | 52.80 | | 7.40 | | 7.14 | 0.00 |
| 4 | % | | 5.61 | | 1.79 | | 3.13 | 0.00 |
| 5 | % | | 12.15 | | 4.13 | | 2.94 | 0.00 |

BIC = **-1907.80*** (N=458) AIC = -1876.84 L= -1861.84

**Six classes:** **total impact of undesirable events**

| Group | Parameter | Standard Estimate | | Standard Error | | T for HO:  Parameter=0 | | Prob > \|T\| |
| --- | --- | --- | --- | --- | --- | --- | --- | --- |
| 1 | Intercept  Linear | 3.85  -0.99 | | 0.49  0.19 | | 7.91  -5.33 | | 0.00  0.00 |
| 2 | Intercept  Linear | 3.12  -0.25 | | 0.19  0.05 | | 16.55  -5.19 | | 0.00  0.00 |
| 3 | Intercept  Linear | 3.26  -0.03 | | 0.13  0.03 | | 25.38  -1.07 | | 0.00  0.29 |
| 4 | Intercept  Linear | 5.68  -0.36 | | 0.82  0.18 | | 6.97  -1.97 | | 0.00  0.049 |
| 5 | Intercept  Linear | 7.08  -0.88 | | 0.47  0.12 | | 15.20  -7.26 | | 0.00  0.00 |
| 6 | Intercept  Linear | 4.64  0.04 | | 0.31  0.06 | | 15.14  0.75 | | 0.00  0.46 |
|  | sigma | 1.23 | | 0.04 | | 30.81 | | 0.00 |
| Group Membership |  |  | |  | |  | |  |
| 1 | % | | 5.69 | | 1.79 | | 3.19 | 0.00 |
| 2 | % | | 24.18 | | 6.99 | | 3.46 | 0.00 |
| 3 | % | | 50.87 | | 7.26 | | 7.00 | 0.00 |
| 4 | % | | 3.49 | | 2.85 | | 1.22 | 0.22 |
| 5 | % | | 5.18 | | 1.99 | | 2.61 | 0.01 |
| 6 | % | | 10.59 | | 3.62 | | 2.93 | 0.00 |

BIC = **-1915.70** (N=458) AIC = -1878.55 L= -1860.55

**Five-class model is best fit for total impact of undesirable events determined by the lowest [absolute] BIC.*

**Table 4. Model parameters of total scores for desirable events.**

**Descriptive data for total scores for desirable events**

| LES time point/occasion | Mean | SD | Median | N |
| --- | --- | --- | --- | --- |
| 1 | 5.96 | 3.54 | 6 | 380 |
| 2 | 5.14 | 3.57 | 4 | 233 |
| 3 | 4.77 | 3.68 | 4 | 220 |
| 4 | 4.37 | 3.23 | 4 | 191 |

Maximum Likelihood Estimates

Model: Censored Normal

**Two classes: total scores for desirable events**

| Group | Parameter | | Standard Estimate | Standard Error | | T for HO:  Parameter=0 | | Prob > \|T\| |
| --- | --- | --- | --- | --- | --- | --- | --- | --- |
| 1 | Intercept  Linear | | 1.94  -0.05 | 0.06  0.01 | | 33.63  -5.14 | | 0.00  0.00 |
| 2 | Intercept  Linear | | 2.79  -0.02 | 0.08  0.01 | | 35.71  -1.35 | | 0.00  0.18 |
|  | sigma | | 0.70 | 0.02 | | 37.23 | | 0.00 |
| Group Membership |  |  | |  | |  | |  |
| 1 | % | | 62.72 | | 5.47 | | 11.47 | 0.00 |
| 2 | % | | 37.28 | | 5.47 | | 6.81 | 0.00 |

BIC=Bayesian information criterion, AIC=Akaike information criterion, L=Log likelihood maximum criterion

BIC = **-1262.16** (N=458) AIC = -1249.78 L= -1243.78

**Three classes: total scores for desirable events**

| Group | Parameter | Standard Estimate | Standard Error | | T for HO:  Parameter=0 | | Prob > \|T\| |
| --- | --- | --- | --- | --- | --- | --- | --- |
| 1 | Intercept  Linear | 0.38  0.10 | 0.77  0.17 | | 0.50  0.57 | | 0.62  0.57 |
| 2 | Intercept  Linear | 2.11  -0.06 | 0.07  0.01 | | 30.47  -4.88 | | 0.00  0.00 |
| 3 | Intercept  Linear  Quadratic | 2.88  -0.01  -0.00 | 0.10  0.05  0.01 | | 29.70  -0.10  -0.16 | | 0.00  0.92  0.88 |
|  | sigma | 0.65 | 0.02 | | 33.46 | | 0.00 |
| Group Membership |  |  |  | |  | |  |
| 1 | % | 5.12 | | 3.46 | | 1.48 | 0.14 |
| 2 | % | 64.89 | | 4.41 | | 14.72 | 0.00 |
| 3 | % | 29.99 | | 5.69 | | 5.27 | 0.00 |

BIC = **-1249.18** (N=458) AIC = -1228.55 L = -1218.55

**Four classes: total scores for desirable events**

| Group | Parameter | Standard Estimate | Standard Error | | T for HO:  Parameter=0 | | | Prob > \|T\| |
| --- | --- | --- | --- | --- | --- | --- | --- | --- |
| 1 | Intercept  Linear  Quadratic | -0.84  0.81  -0.07 | 0.34  0.18  0.02 | | -2.45  4.51  -3.43 | | | 0.01  0.00  0.00 |
| 2 | Intercept  Linear  Quadratic | 1.94  -0.48  0.04 | 0.21  0.19  0.02 | | 9.43  -2.55  2.06 | | | 0.00  0.01  0.04 |
| 3 | Intercept  Linear | 2.15  -0.05 | 0.06  0.01 | | 38.27  -4.64 | | | 0.00  0.00 |
| 4 | Intercept | 2.90 | 0.07 | | 43.89 | | | 0.00 |
|  | sigma | 0.62 | 0.02 | | 35.31 | | | 0.00 |
| Group Membership |  |  |  | |  | | |  |
| 1 | % | 3.31 | | 0.93 | | 3.578 | 0.00 | |
| 2 | % | 8.42 | | 4.63 | | 1.818 | 0.07 | |
| 3 | % | 61.01 | | 5.03 | | 12.141 | 0.00 | |
| 4 | % | 27.25 | | 4.19 | | 6.504 | 0.00 | |

BIC = **-1238.37*** (N=458) AIC = -1211.54 L = -1198.54

**Four-class model is best fit for total number of desirable events determined by the lowest [absolute] BIC.*

**Table 5. Model parameters of impact scores for desirable events.**

**Descriptive data of impact scores for desirable events**

| LES time point/occasion | Mean | SD | Median | N |
| --- | --- | --- | --- | --- |
| 1 | 19.03 | 12.74 | 16 | 380 |
| 2 | 16.16 | 12.94 | 13 | 233 |
| 3 | 13.54 | 12.38 | 10 | 220 |
| 4 | 13.04 | 10.81 | 10.5 | 191 |

Maximum Likelihood Estimates

Model: Censored Normal *transformed*

**Four classes: impact scores for desirable events**

| Group | Parameter | Standard Estimate | Standard Error | | T for HO:  Parameter=0 | | Prob > \|T\| |
| --- | --- | --- | --- | --- | --- | --- | --- |
| 1 | Intercept  Linear  Quadratic | -1.28  1.16  -0.09 | 0.92  0.45  0.04 | | -1.40  2.58  -2.13 | | 0.16  0.01  0.03 |
| 2 | Intercept  Linear  Quadratic | 3.60  -0.36  0.03 | 0.13  0.07  0.01 | | 28.84  -4.80  3.45 | | 0.00  0.00  0.00 |
| 3 | Intercept  Linear | 4.54  -0.05 | 0.28  0.03 | | 16.24  -1.85 | | 0.00  0.06 |
| 4 | Intercept | 5.88 | 0.32 | | 18.20 | | 0.00 |
|  | sigma | 1.21 | 0.03 | | 35.06 | | 0.00 |
| Group Membership |  |  |  | |  | |  |
| 1 | % | 3.43 | | 1.03 | | 3.32 | 0.00 |
| 2 | % | 52.07 | | 8.36 | | 6.23 | 0.00 |
| 3 | % | 34.82 | | 6.71 | | 5.19 | 0.00 |
| 4 | % | 9.68 | | 5.07 | | 1.91 | 0.06 |

BIC=Bayesian information criterion, AIC=Akaike information criterion, L=Log likelihood maximum criterion

BIC = **-1894.34** (N=458) AIC = -1867.52 L = -1854.52

**Five classes: impact scores for desirable events**

| Group | Parameter | Standard Estimate | Standard Error | | T for HO:  Parameter=0 | | Prob > \|T\| |
| --- | --- | --- | --- | --- | --- | --- | --- |
| 1 | Intercept  Linear  Quadratic | -1.41  1.20  -0.09 | 0.75  0.38  0.04 | | -1.87  3.20  -2.39 | | 0.06  0.00  0.02 |
| 2 | Intercept  Linear  Quadratic | 3.79  -0.31  0.02 | 0.13  0.07  0.01 | | 28.71  -4.52  3.13 | | 0.00  0.00  0.00 |
| 3 | Intercept  Linear  Quadratic | 2.56  -0.44  0.03 | 0.44  0.37  0.04 | | 5.85  -1.18  0.70 | | 0.00  0.24  0.49 |
| 4 | Intercept  Linear | 4.83  -0.04 | 0.28  0.03 | | 17.27  -1.43 | | 0.00  0.15 |
| 5 | Intercept | 6.07 | 0.35 | | 17.27 | | 0.00 |
|  | sigma | 1.17 | 0.04 | | 33.00 | | 0.00 |
| Group Membership |  |  |  | |  | |  |
| 1 | % | 3.25 | | 0.99 | | 3.29 | 0.00 |
| 2 | % | 55.94 | | 5.92 | | 9.46 | 0.00 |
| 3 | % | 6.29 | | 3.65 | | 1.72 | 0.09 |
| 4 | % | 27.67 | | 5.50 | | 5.03 | 0.00 |
| 5 | % | 6.85 | | 4.34 | | 1.58 | 0.12 |

BIC = **-1899.54** (N=458) AIC = -1864.47 L = -1847.47

**Three classes: impact scores for desirable events**

| Group | Parameter | Standard Estimate | Standard Error | | T for HO:  Parameter=0 | | Prob > \|T\| |
| --- | --- | --- | --- | --- | --- | --- | --- |
| 1 | Intercept  Linear | 1.01  0.07 | 0.45  0.07 | | 2.25  1.01 | | 0.03  0.31 |
| 2 | Intercept  Linear | 3.72  -0.11 | 0.11  0.02 | | 35.18  -6.74 | | 0.00  0.00 |
| 3 | Intercept  Linear | 5.33  -0.04 | 0.17  0.03 | | 31.76  -1.69 | | 0.00  0.09 |
|  | sigma | 1.24 | 0.04 | | 35.69 | | 0.00 |
| Group Membership |  |  |  | |  | |  |
| 1 | % | 7.46 | | 2.38 | | 3.13 | 0.00 |
| 2 | % | 65.32 | | 4.41 | | 14.83 | 0.00 |
| 3 | % | 27.23 | | 4.43 | | 6.14 | 0.00 |

BIC = **-1893.24*** (N=458) AIC = -1874.67 L = -1865.67

**Three-class model is best fit for impact score of desirable events determined by the lowest [absolute] BIC.*
