# Supplementary material for: Stressful life events during the perimenopause: longitudinal observations from the seattle midlife women’s health study
Source: Womens Midlife Health. 2023 Sep 5;9:6. doi: 10.1186/s40695-023-00089-y (PMC10478480; doi:10.1186/s40695-023-00089-y)
Supplement: Supplementary file 2 — Supplementary Material 2 [file 40695_2023_89_MOESM2_ESM.docx]

**Responses to Reviewers of Women’s Midlife Health**

**WMLH D2300004R1**

**Stressful life events during the perimenopause: longitudinal observations from the Seattle Midlife Women’s Health Study**

**08132023**

| **Reviewer’s comments** | **Revision** |
| --- | --- |
| Review #2 |  |
| *Abstract*  "The purpose of this study was … to identify predictors of socio-economic factors and demographic characteristics."  That sounds like the outcome variables were socio-economic factors and demographic characteristics.  In the methods, the analytic strategies identify predictors for each group-based trajectory model. So, the model itself is the outcome.  In the results, the outcome is SLEs. I think the abstract could be rewritten to make the outcome(s) of interest more clear and consistent.  In the abstract background, "(SLE)" should be added the first time "stressful life events" is used because "SLE" is used later on. In the abstract conclusion, "the stages" should be "menopausal stages" for clarity.  *Methods*  I recommend just picking "occasions" or "time points" (once you make it clear that "occasion" means time point). It's OK as is, but there is no need to use both terms each time.  At the very end of the Methods section, the authors say that multinomial logistic regression analysis was used to identify predictors of the LES trajectories using baseline data. To me, that is the most clear description of the outcome. The outcomes are the LES trajectories.  I recommend that the tables for model parameters be put into supplementary tables. | Changed to “The purpose of this study was to describe the longitudinal patterns of SLEs of women during midlife and to identify predictors of the SLE trajectories using baseline data.”  The outcome variable/dependent variable are the models. The independent variables are the SLE scores and the predictors of socio-economic factors and demographic characteristics.  Changed the results section to read, “Socio-economic factors, demographic variables, and menopausal transition stages were not significant predictors of any of the four GBTMs.”  (SLE) was added in the abstract background after the first use of stressful life events.  Stages were changed to menopausal stages.  *Methods*  Occasion/Time point was used once, and then “time points” was used throughout the paper for clarity.  Thank you.  I am fine with the Tables for Model Parameters being placed into supplementary material. |
| Reviewer #3 |  |
| New issues: Page 12, top to middle of page 13. Here authors present some descriptive statistics for the study sample. These should be moved to the results section of the manuscript. | The descriptive statistics of the study sample (see Table 1) were moved to the Results section. |
